# Functional movement assessment by means of inertial sensor technology to discriminate between movement behaviour of healthy controls and persons with knee osteoarthritis

**DOI:** 10.1186/s12984-020-00694-2

**Published:** 2020-05-19

**Authors:** Rob van der Straaten, Mariska Wesseling, Ilse Jonkers, Benedicte Vanwanseele, Amber K. B. D. Bruijnes, Jan Malcorps, Johan Bellemans, Jan Truijen, Liesbet De Baets, Annick Timmermans

**Affiliations:** 1 REVAL Rehabilitation Research Center, Hasselt University, Diepenbeek, Belgium; 2 Department of Movement Sciences, Human Movement Biomechanics, KU Leuven, Leuven, Belgium; 3 Department of Orthopaedics, Ziekenhuis Oost-Limburg, Genk, Belgium; 4 Department of Orthopaedic Surgery, Jessa Hospital, Hasselt, Belgium

**Keywords:** Inertial sensors, Motion-analysis, Ambulatory, Validation, Repeatability, Functional movement, Questionnaires, Osteoarthritis, Pain, Fear of movement

## Abstract

**Background:**

Apart from biomechanical alterations in movement patterns, it is known that movement limitations in persons with knee osteoarthritis (PwKOA) are related to an individual’s perception and belief regarding pain and disability. To gain more insights into the functional movement behaviour of PwKOA in a clinical setting, inertial sensor technology can be applied. This study first aims to evaluate the ability of inertial sensors to discriminate between healthy controls (HC) and PwKOA. Secondly, this study aims to determine the relationship between movement behaviour, pain-related factors and disability scores.

**Methods:**

Twelve HC and 19 PwKOA were included. Five repetitions of six functional movement tasks (walking, forward lunge, sideward lunge, ascent and descent stairs, single leg squat and sit-to-stand) were simultaneously recorded by the inertial sensor system and a camera-based motion analysis system. Statistically significant differences in angular waveforms of the trunk, pelvis and lower limb joints between HC and PwKOA were determined using one-dimensional statistical parametric mapping (SPM1D). The Knee injury and Osteoarthritis Outcome Score and TAMPA scale for Kinesiophobia were used to evaluate the relationship between discriminating joint motion, pain-related factors and disability using spearman’s correlation coefficients.

**Results:**

PwKOA had significantly less trunk rotation, internal pelvis rotation and knee flexion ROM during walking. Additionally, the reduced knee flexion (i.e. at the end of the stance phase and swing phase) was related to increased level of perceived pain. During the sideward lunge, PwKOA had significantly less knee flexion, ankle plantarflexion and hip abduction. This decreased hip abduction (i.e. during stance) was related to higher fear of movement. Finally, PwKOA had significantly less knee flexion during the forward lunge, single leg squat and during ascent and descent stairs. No significant correlations were observed with disability.

**Conclusions:**

Inertial sensors were able to discriminate between movement characteristics of PwKOA and HC. Additionally, significant relationships were found between joint motion, perceived pain and fear of movement. Since inertial sensors can be used outside the laboratory setting, these results are promising as they indicate the ability to evaluate movement deviations. Further research is required to enable measurements of small movement deviations in clinically relevant tasks.

## Introduction

Knee osteoarthritis (KOA) is a common degenerative disease and one of the leading causes of disability in elderly persons [1]. Above 60 years, 10% of the males and 18% of the females show symptoms of KOA, including muscle weakness, reduced range of motion (ROM), loss of proprioception and altered joint loading [2]. Together, these factors lead to a reduced joint function, which results in the development of pain, functional limitations and loss of mobility [3]. Pain intensity, functioning and disability are typically evaluated by patient reported outcome measures (PROMs). These self-reported measures are disease-specific, convenient to use and easy to incorporate in clinical practice to monitor treatment effects [4]. However, PROMs are subjective, potentially affected by pain-related beliefs and often suffer from ceiling effects, which may mask limitations in the actual functioning of an individual [5].

Objective measures of joint kinematics, assessed during the execution of functional tasks, might therefore be of interest to objectively evaluate the actual performance. Objective motion analysis, which enables the measurement of segmental movement in three dimensions, is generally performed with high precision in a movement laboratory [6]. However, as these measurements are expensive, complex and time consuming, they are not regularly available in clinical practice. Over the last decades, inertial sensor technology has gained potential to be used for three-dimensional motion analysis, as it is easy to use in a clinical setting and is far less time consuming then camera-based motion analysis (i.e. in a laboratory) [7]. However, in order to confidently use inertial sensor technology in clinical practice its reliability and validity needs to be confirmed. So far, good reliability and construct validity were reported for lower extremity joint angles during level walking [8–11]. However, when assessing more demanding tasks (such as a forward lunge, squat, vertical jump or stair ascent and descent), reliability and construct validity decreased, especially for frontal and transverse plane angles [12–16]. Furthermore, these studies determined the reliability and validity based on distinct points within the waveform (i.e. peak values or ROM) or from a selected phase in the waveform (e.g. swing, stance), thereby losing information about the kinematic waveform [17]. In addition, abovementioned studies only included healthy participants, for whom these demanding tasks impose no difficulties. As such, it is also unknown whether kinematic waveforms obtained by means of inertial sensor technology have appropriate discriminant validity to differentiate between joint motion of healthy controls (HC) and persons with KOA (PwKOA).

It is believed that the degree of movement disability is not only related to changes in biomechanics associated with KOA, but also to the individuals’ perceived level of disability and their perception of pain [18]. Therefore, it is important to assess the relationship between the kinematic outcome of inertial sensors and the outcome on disability and pain related beliefs by PROMs in PwKOA. Few studies have investigated the relationship between biomechanical changes of walking and the perceived level of pain and disability [3, 4, 19]. Despite the fact that the reported correlations were low, significant correlations between self-reported levels of function and pain and knee ROM, walking speed and stride length were found in PwKOA [4, 5]. Furthermore, it was reported that knee flexion ROM, hip extension ROM and external rotation ROM during walking were determinants of disability in PwKOA [3]. However, as it is proposed that (mal)adaptive movement strategies in KOA are more prominent during challenging and demanding tasks [6], it is of interest to assess the relation between perceived knee function, pain-related factors and objective motion analysis during the performance of these challenging tasks.

Therefore, the primary aim of this study is to evaluate which of the trunk, pelvis and lower limb angular waveforms differentiate between HC and PwKOA during functional movement tasks, based on the camera-based system and the inertial sensor system. As a secondary aim, this paper evaluates the relationship between these discriminating objective parameters and the individuals’ perceived level of function and pain-related factors.

## Methods

### Participants

Twelve healthy volunteers, who were recruited from a local network of seniors and relatives and 19 persons with unilateral KOA, who were recruited from two local hospitals (Jessa Hospital Hasselt, Belgium and Ziekenhuis Oost Limburg Genk, Belgium), participated in the study. It was a conscious decision to select only 12 healthy volunteers, as both legs were included for analysis, which were compared to the affected leg of the 19 PwKOA.

Healthy participants were included if they were between 50 and 75 years old, able to walk 10 m, able to ascent and descent a staircase of four steps, and able to understand the Dutch language. Exclusion criteria were pain or pathology in the torso or lower limb joints, or any systemic or neurological disease.

PwKOA were included if they were between 50 and 75 years old, received a diagnosis of end-stage unilateral KOA and were awaiting for a total knee replacement surgery. Furthermore, they had to be able to walk 10 m, able to ascent and descent a staircase of four steps, and to understand the Dutch language. Exclusion criteria were a corticosteroid injection in the knee (up to 3 months before the inclusion), diagnosis of degenerative disorders in other lower limb joints, neurological conditions or a history of pathological osteoporotic fractures.

### Data collection

For the present study, both HC and PwKOA performed six movement tasks including walking, forward lunge, sideward lunge, ascent and descent stairs, single leg squat and sit-to-stand. Five repetitions of all tasks were simultaneously recorded by the camera-based system (Vicon, Oxford Metrics, Oxford, UK) and the inertial sensor system (MVN BIOMECH Awinda, Xsens Technologies, Enschede, The Netherlands). The instructions to the participant are presented in Table 1. All tasks were performed barefoot and were practiced to familiarize and to make sure they were executed according to the instructions. The tasks were explained to the participant and were showed by the operator that was guiding the measurements. The number of times the participant practiced was not registered, given that this was not the scope of the research. On average between 1 and 5 practice trials were necessary to get the participants familiarized with the requested movement. All participants were able to rest in between repetitions if required.

**Table 1:** Detailed description of the instructions to the participants

Task	Instruction to participant
Walking	Start in an upright position, with the feet aligned to the marked starting line. Walk at comfortable speed, as you would normally do, to the other side of the lab (10 m) until you have passed the stopping line.
Forward lunge	Start in an upright position, keep your hands slightly away from your body and the toes aligned with the marked starting line. Step forward with your heel over the predetermined distance (70% leg length) as marked on the ground. While stepping forward, bring the weight of your upper body over the leading leg and be sure that the contralateral leg keeps in contact with the floor throughout the forward lunge. Make sure that the entire foot contacts the ground and subsequently step backwards to the initial start position.
Sideward lunge	Start in an upright position, keep your hands slightly away from your body and the side of the foot aligned with the marked starting line. Step sideward with your foot over the predetermined distance (70% leg length) as marked on the ground and keep the foot parallel to the marked line. While stepping sideward, bring the weight of your upper body over the leading leg and be sure that the contralateral leg keeps in contact with the floor throughout the sideward lunge. Make sure that the entire foot makes contact with the ground and subsequently step backwards to the initial start position.
Ascent / Descent stairs	Start in an upright position, with the feet aligned next to each other in front of the first step. Ascent the stairs and wait on top of the staircase until we have given the instruction to turn around. At our command, descent the stairs and wait at the bottom of the stair until you are instructed to turn around.
Single leg squat	Stand still with feet shoulder width apart and put your hands on the pelvis. Shift the weight to one side (i.e. stand on one leg), lift the other foot from the ground. When standing on one leg, squat on the standing leg as deep as possible but remain balanced and make sure the other leg is not contacting the ground. When maximal flexion is reached, extend the knee and when the leg is fully extended, place your other foot down again.
Sit to Stand	Stand with your back towards the stool with the feet shoulder width apart and with the arms hanging alongside of your body. Sit down without looking over your shoulder, remain seated (similar as you would sit on a chair), and stand up again (without swinging your arms). The stool height was pre-set on the participants knee height.

As this study is part of a larger of a larger cohort study, justification of the number of subjects is based on an overview of compartmental forces measured in participants that received TKR [20]. Fregly and colleagues reported an average medial compartmental force of 1.61 (±0.305) body weight during gait. Assuming an increase of 1 Stdev (0.31 BW) to be clinically significant in participants that suffer from medial compartmental OA, a sample of 14 participants was calculated with a of 0.05 and power level of 0.80. To overcome the problem that participants could dropout after inclusion some additional participants were recruited.

#### Inertial sensor system

**Fig. 1:**
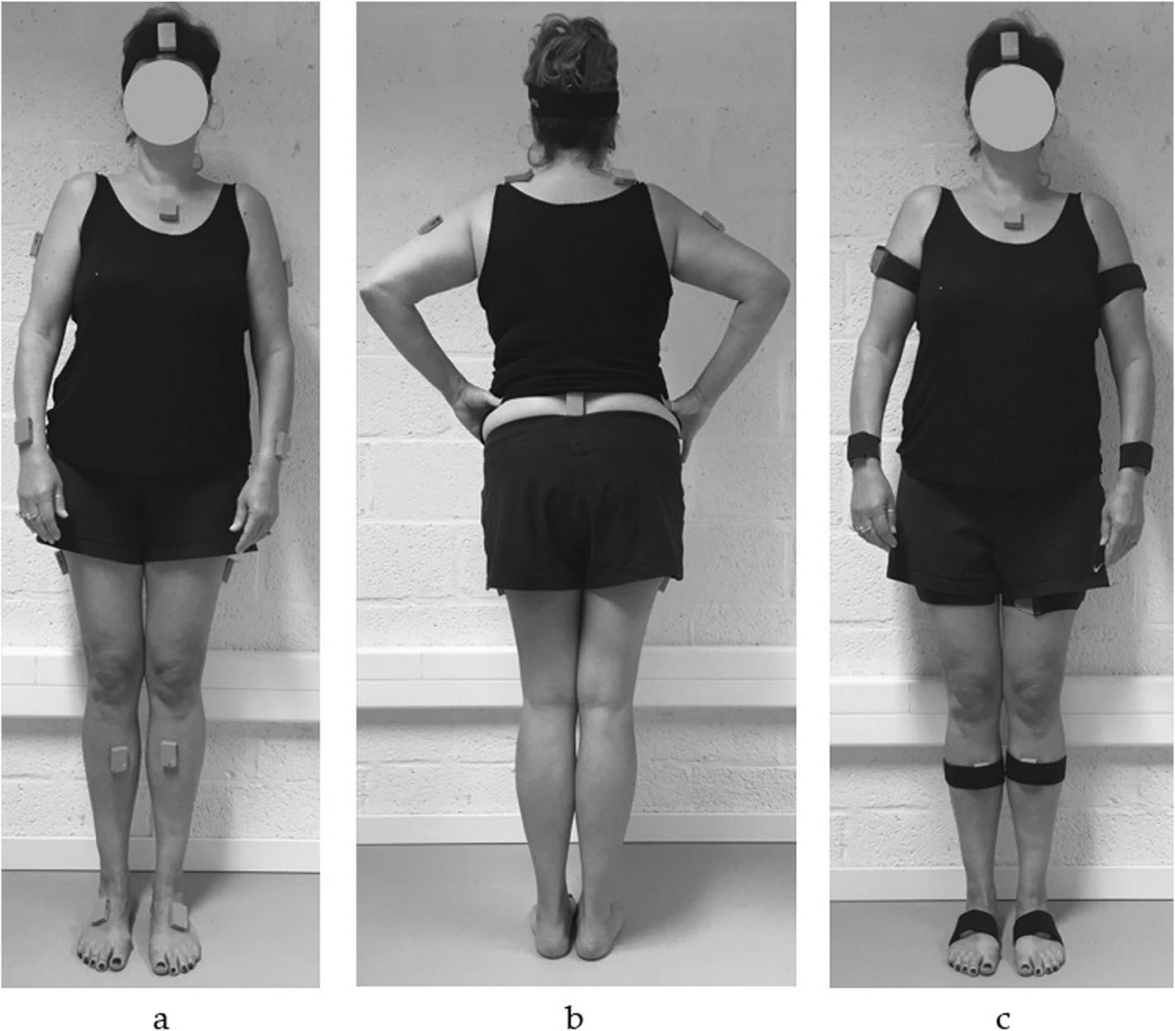
Positioning of inertial sensors in **a** anterior and **b** posterior view, and **c** anterior view with the straps to minimize soft tissue artefacts

Three-dimensional joint kinematics of the trunk, pelvis and lower limb joints were measured using 15 inertial sensors (MVN BIOMECH Awinda). The inertial sensors were positioned according to the guidelines of the manufacturer, i.e. on the dorsal side of the foot, the medial surface of the tibia (just underneath the tibial tuberosity), laterally on the thigh, on L5/S1, the dorsal side of the forearm (distally between ulna and radius), on the upper arm slight dorsal of the middle line, along the superior border of the scapulae, on the flat part of the sternum, and on the forehead [21]. The sensors were positioned on the skin using double-sided adhesive tape and were secured with a strap to minimize soft-tissue artefacts (Fig. 1). Joint kinematics were recorded using the MVN BIOMECH software (60 Hz, MVN Studio 4.4, firmware version 4.3.1). The participants’ body dimensions were measured in order to scale the model and a static calibration (N-pose) was performed in order to align the sensors to the body segments. Three-dimensional joint kinematics were directly derived from the MVN software, which are defined according to the recommendations of the international society of biomechanics [22].

#### Optoelectronic system

Sixty-five reflective markers were positioned, according to the Plug-in-Gait model, with additional markers positioned on the sacrum, medial femur epicondyles, medial malleoli and marker clusters on the upper and lower legs and arms [23]. Three-dimensional marker trajectories were recorded using a 10 camera VICON System (100 Hz, Oxford Metrics, Oxford, UK). A musculoskeletal model with 6 degrees of freedom (DOF) in the tibiofemoral joints, 6 DOF for the pelvis, 3 DOF for the trunk and hip joint and 1 DOF for the ankle joint was used to process the data [24]. The musculoskeletal model was implemented in SIMM (Motion Analysis Corporation, Santa Rosa, CA), using the Dynamics Pipeline (Symbolic Dynamics, Inc., Mountain View, CA) and SD/Fast (PTC, Needham, MA) to generate the multibody equations of motion [25]. To scale the anthropometry and mass of the participant, a generic model was used. Inverse kinematics were used to calculate full body joint kinematics [26].

#### Patient-reported outcome measures

The *Knee injury and Osteoarthritis Outcome Score (KOOS)* evaluates symptoms and functions in individuals with knee injury and osteoarthritis [7]. The KOOS consists of 5 subscales: pain, other symptoms, function in daily living (ADL), function in sports and recreation (Sports/Rec) and knee related quality of life (QOL). Each subscale is scored between 0 and 100, where 0 indicates extreme symptoms and 100 indicates no symptoms. The KOOS has adequate reliability and validity to be used for individuals with knee injuries and knee osteoarthritis [27]. Within the present study, the subscales pain and ADL were reported as measures of perceived pain and disability.

The *TAMPA scale for Kinesiophobia (TSK)* evaluates pain-related fear of movement [28]. It consists of 17 questions that are scored from 1 to 4 (total range 17–68). Scores greater than 37 are accepted as a clinical threshold for fear of movement, with higher scores indicating a higher level of kinesiophobia [29, 30]. The TSK was shown to be valid and reliable [31–33]. Both HC and PwKOA completed the KOOS and PwKOA completed the TSK, before the measurement session started.

### Data analysis

For both systems, the joint kinematics were time normalized from 0 to 100%, using a custom written algorithm in Matlab (2016b, Mathworks, Inc., Natick, MA, USA). For walking, a stride was normalized from heel-strike to heel-strike. The forward lunge and sideward lunge were normalized from the period that the foot was lifted more than 2 cm above the floor at the start and the end of both lunges. For ascending and descending stairs, the stance and swing phase (i.e. the period that the foot contacted the stair-step until the foot was positioned back on the next stair-step), was normalized. The single leg squat was normalized for the period in which the contralateral foot was more than 2 cm lifted from the ground and the sit to stand task was normalized from the period that the trunk or pelvis was moving down. Furthermore, the trunk and pelvic angles of the inertial sensor system were transformed to account for differences in the segment coordinate frames in the underlying kinematic models between systems. Within the musculoskeletal model the trunk was defined as one rigid body, while the trunk was divided into four segments in the MVN BIOMECH model. Therefore, these segments were accumulated in order to compare both models in both models. Additionally, the MVN BIOMECH pelvic orientation was converted into Euler angles to match the pelvic angles of the musculoskeletal model (expressed in the global reference frame).

#### Discriminant validity

Based on one-dimensional statistical parametric mapping (SPM1D) analysis, the entire waveforms were compared between the HC and PwKOA. Depending on the normality of the angular waveform (i.e. assessed by using the SPM normality function for a two-sample t-test), a parametric two-sample t-test (SPM{t}, α = 0.05) or a non-parametric two-sample t-test (SnPM{t}, α = 0.05) was applied in order to evaluate the discriminant validity. If a significant difference was present within the waveform, SPM provided a *p*-value for each time the threshold of significance was exceeded. The camera-based system was used as a reference and only those kinematics differences that were identified by both systems were reported. The discriminating differences of the inertial sensor system were only reported for waveforms with acceptable construct validity, and if the difference was greater than the minimum detectable change (MDC).

More information regarding the reliability, agreement and construct validity of the discriminating angular waveforms is provided in the supplementary materials.

#### Relationship of joint motion with patient reported outcome measures

In case discriminant validity was confirmed, the ROM of the specific joint motion measured by the inertial sensor system (e.g. knee flexion ROM) was determined in PwKOA over the period significant differences were present. These ROMs were used to assess the relationship with KOOS pain, KOOS ADL and TSK, using a Spearman’s (rho) correlation.

## Results

### Participants

For the present study, 12 HC and 19 persons with unilateral KOA (Kellgren / Lawrence (KL) grade 3 (*n* = 1) and grade 4 (*n* = 18)) participated. PwKOA were significantly older (*p* = 0.02) compared to the HC (Table 2). No other significant differences were observed between groups in height, weight or BMI. The PROMs show that HC had significantly lower KOOS subscales, confirming that there were no indications for knee related pain or disability in the HC. Furthermore, PwKOA showed fear of movement, as the TSK score was greater than the clinical threshold (i.e. > 37).

**Table 2:** Participant characteristics (mean ± SD)

	HC (*n* = 12)	PwKOA (*n* = 19)
Male / Female	6/6	12/7
Age (years)	59.8 (± 7.0)	65.1 (± 5.2) *
Height (m)	1.71 (± 0.10)	1.75 (± 0.08)
Weight (kg)	74.3 (± 14.9)	79.8 (± 8.4)
BMI	25.1 (± 3.4)	26.0 (± 2.2)
M / L / T compartment KOA	–	7 / 8 / 4
**Questionnaires**		
KOOS Pain	95.1 (± 7.0)	50.9 (± 12.2) *
KOOS Symptoms	98.5 (± 3.6)	52.3 (± 18.4) *
KOOS ADL	98.7 (± 2.9)	56.4 (± 15.9) *
KOOS Sport/Rec	94.6 (± 7.8)	24.1 (± 23.9) *
KOOS QOL	94.8 (± 6.4)	28.0 (± 16.3) *
TSK	–	38.5 (± 6.9)

* Significant difference between HC and PwKOA (*p* < 0.05)

*BMI* Body Mass Index, Medial- / Lateral- / Tricompartmental KOA, *KOOS ADL* functioning during daily living, *KOOS Sport/Rec* functioning during recreation or sports, *KOOS QOL* knee related quality of life.

### Discriminant validity

**Fig. 2:**
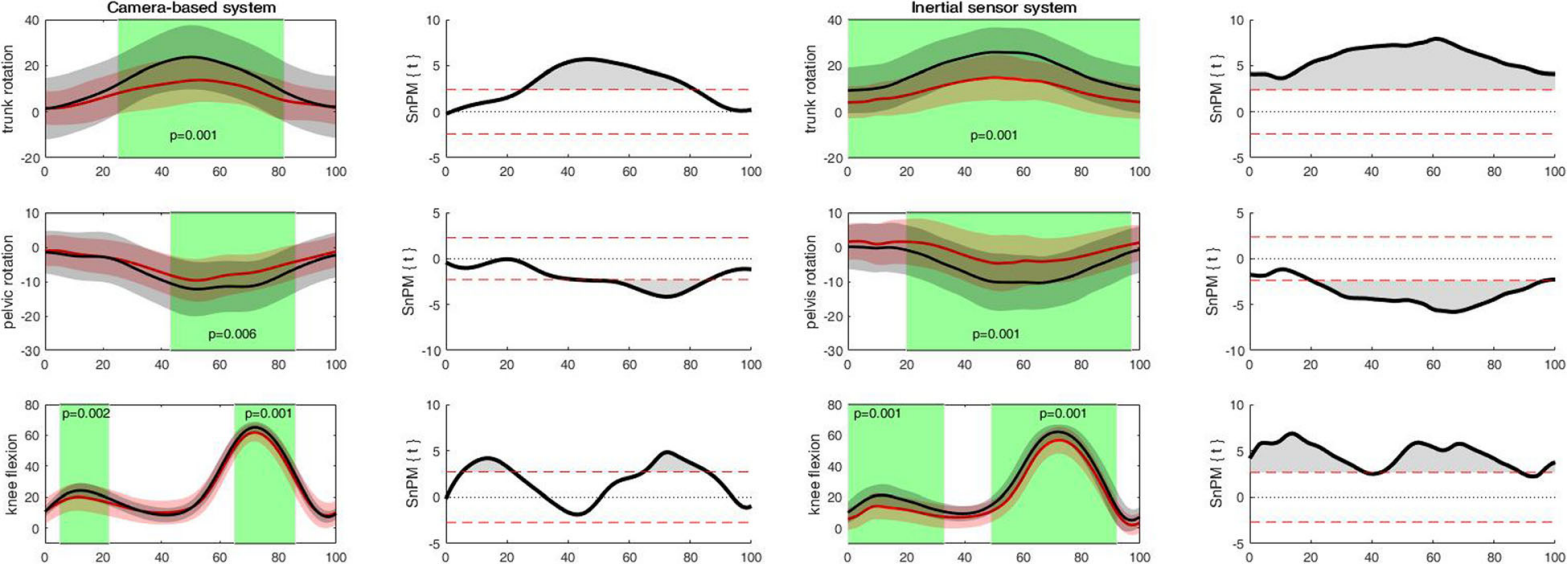
Discriminant angular waveforms (mean ± SD) between HC (red) and PwKOA (red) during walking

**Fig. 3:**
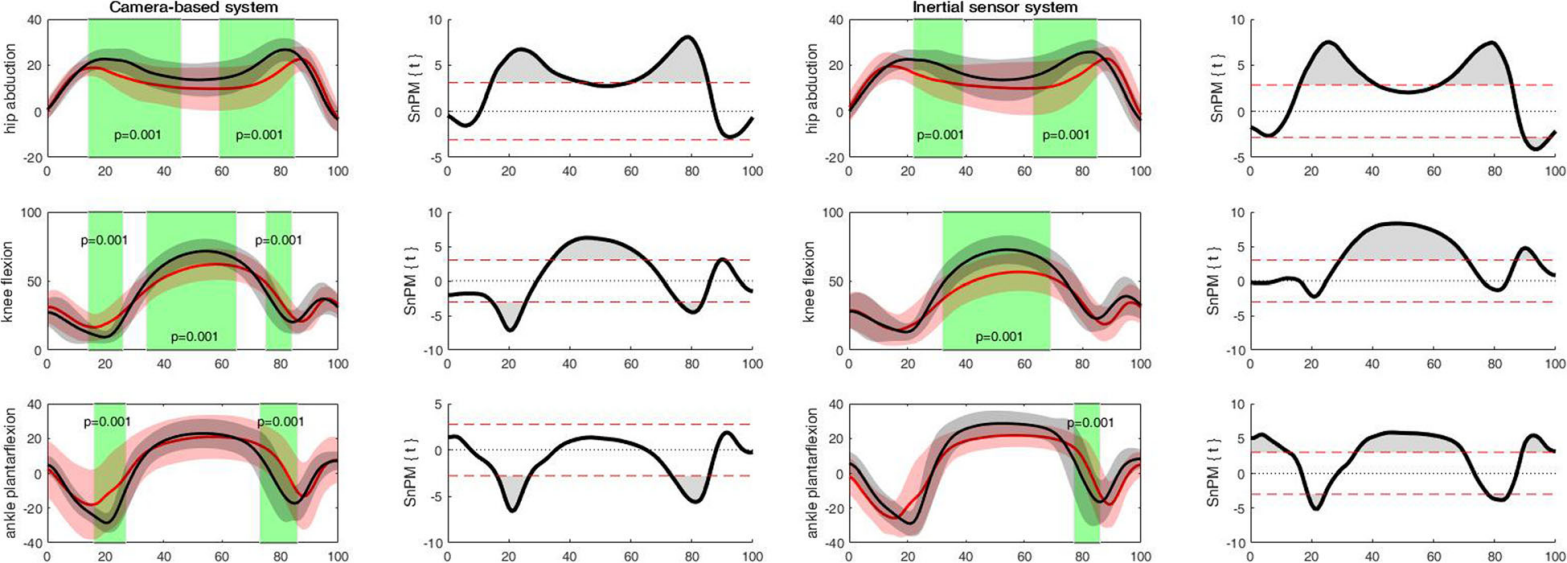
Discriminant angular waveforms (mean ± SD) between HC (red) and PwKOA (red) during the sideward lunge. The shaded areas within the waveforms represent the area where the angular waveforms are significantly different (i.e. in which the SPM{t} / SnPM{t} exceeds the critical threshold).

**Fig. 4:**
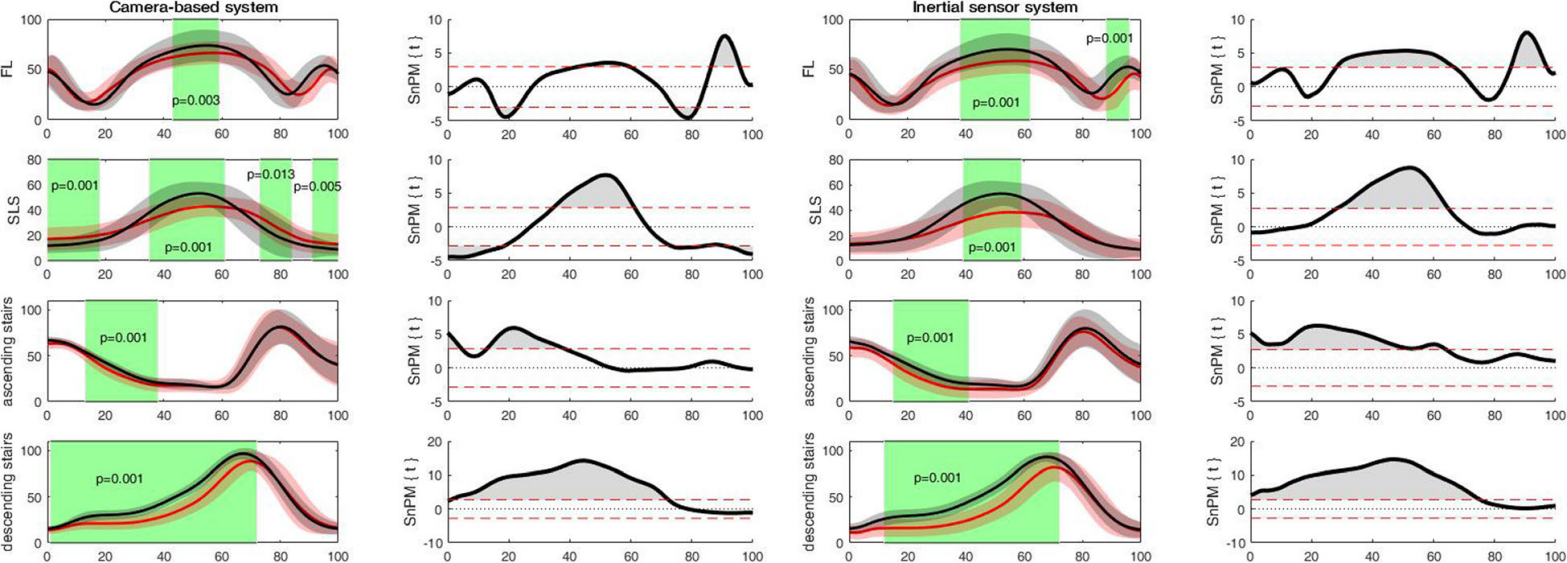
Discriminant angular waveforms (mean ± SD) between HC (red) and PwKOA (red) during the forward lunge (FL), single leg squat (SLS), and ascending/descending stairs The shaded areas within the waveform represent the area where the angular waveforms are significantly different (i.e. in which the SPM{t} / SnPM{t} exceeds the critical threshold).

For the camera-based system, discriminating angular waveforms between HC and PwKOA were found for all tasks and most of the angular waveforms (Table 3). Based on these differences, the discriminating angular waveforms of the inertial sensor system were selected (as the camera-based system was used as a reference). For the inertial sensor system, discriminating differences were found in all tasks, except for the sit to stand task, and only for specific angular waveforms (Table 3). Accordingly for walking, PwKOA showed significantly less *trunk rotation* for the entire stride (0 to 100%; *p* = 0.001), less *internal pelvic rotation* ROM during the transition from stance to swing phase (39 to 80%; *p* = 0.001) of the stride and reduced *knee flexion* ROM in the stance (0 to 33%; *p* = 0.001) and swing phase (49 to 92%; *p* = 0.001) of the stride, compared to HC (Fig. 2). For the more demanding and challenging tasks, discriminating differences were observed in all tasks except the sit to stand task. For the forward lunge, PwKOA had significantly less *knee flexion* ROM from 38 to 62% (*p* = 0.001) and from 88 to 96% (p = 0.001). During the sideward lunge PwKOA had significantly less *hip abduction* ROM from 22 to 39% (*p* = 0.001) and from 63 to 85% (p = 0.001), less *knee flexion* ROM from 32 to 69% (*p* = 0.001) and less *ankle plantarflexion* ROM from 21 to 25% and 79 to 85% (*p* = 0.001) of the sideward lunge (Fig. 3). For the single leg squat, PwKOA had significantly less *knee flexion* ROM from 39 to 59% (*p* = 0.001) of the task. For ascending stairs, PwKOA had significantly less *knee flexion* ROM from 15 to 41% (*p* = 0.001), and for descending stairs less *knee flexion* ROM from 12 to 72% (*p* = 0.001) of the task (Fig. 4). It should be noted that part of the data could not be used, i.e. due to technical errors or because not all markers were visible. The number of repetitions that were used for the analysis are provided in Table 3, which correspond with 75% of the data from all trials that were recorded.

**Table 3:** Overview of discriminating differences in camera-based system and inertial sensor system

		Walk	Forward lunge	Sidward lunge	Sit to Stance	Single leg Squat	Ascent stairs	Descent stairs
#Trials HC;PwKOA		101; 87	91; 79	78; 61	92; 52	99; 68	96; 69	98; 62
frontal plane	trunk	cs	cs	cs	cs	ns	cs	ns
	pelvis	cs	cs	cs	cs	ns	ns	cs
	hip	cs	cs	**both**	ns	ns	cs	ns
	knee	ns	cs	cs	cs	cs	cs	cs
Transverse plane	trunk	**both**	cs	cs	cs	cs	cs	ns
	pelvis	**both**	cs	cs	cs	ns	cs	cs
	hip	ns	cs	cs	ns	ns	ns	cs
	knee	cs	cs	cs	cs	ns	cs	cs
Sagittal plane	trunk	cs	cs	cs	cs	cs	cs	cs
	pelvis	ns	cs	cs	cs	ns	cs	cs
	hip	cs	cs	cs	ns	cs	cs	cs
	knee	**both**	**both**	**both**	ns	**both**	**both**	**both**
	ankle	cs	cs	**both**	cs	cs	cs	cs

*ns* no significant differences, *cs* discriminating differences for camera-based system; both: corresponding discriminating differences in both systems; #Trials HC; PwKO: number of trials used for analysis in both populations.

The shaded areas within the waveforms represent the area where the angular waveforms are significantly different (i.e. in which the SPM{t} / SnPM{t} exceeds the critical threshold).

#### Correlation with PROMs

Correlations between PROMs and joint ROM (measured by the inertial sensor system) were only performed on the data from the PwKOA (Table 4). Only two significant correlations were observed, i.e. less knee flexion ROM in the last part of the stride (49–92%) of walking was related to more perceived pain, and less hip abduction during the first part of the sideward lunge (22–39%), i.e. around initial ground contact, was related to more fear of movement. No significant correlations were observed between joint ROM and disability.

**Table 4:** Significant correlations between discriminant joint kinematics and PROMs

Joint ROM	Correlation	KOOS pain	KOOS ADL	TSK
Walk trunk rotation	rho	−0.046	0.053	−0.444
(0–100%)	*p*-value	0.857	0.834	0.065
Walk pelvis rotation	rho	0.288	0.211	−0.395
(0–39%)	*p*-value	0.246	0.400	0.104
Walk knee flexion	rho	0.357	0.155	−0.084
(0–33%)	*p*-value	0.146	0.540	0.740
Walk knee flexion	rho	**0.536**	−0.008	0.271
(49–92%)	*p*-value	**0.022**	0.974	0.277
Forward lunge knee flexion	rho	−0.118	0.055	0.211
(38–62%)	*p*-value	0.641	0.829	0.400
forward lunge knee flexion	rho	0.305	−0.008	−0.122
(85–97%)	*p*-value	0.218	0.976	0.629
Sideward lunge hip abduction	rho	0.229	0.169	**−0.575**
(22–39%)	*p*-value	0.393	0.531	**0.020**
Sideward lunge hip abduction	rho	−0.056	− 0.302	− 0.250
(63–85%)	*p*-value	0.836	0.256	0.351
Sideward lunge knee flexion	rho	0.012	0.078	−0.055
(32–69%)	*p*-value	0.965	0.774	0.841
Sideward lunge ankle flexion	rho	0.194	−0.169	0.232
(79–85%)	*p*-value	0.472	0.531	0.387
Single leg squat knee flexion	rho	0.155	0.212	−0.031
(39–59%)	*p*-value	0.553	0.414	0.905
Ascending stairs knee flexion	rho	0.324	0.225	0.170
(15–41%)	*p*-value	0.221	0.401	0.529
Descending stairs knee flexion	rho	0.402	0.219	0.496
(12–72%)	*p*-value	0.123	0.415	0.051

## Discussion

The primary objective of the present study was to examine which angular waveforms differentiate between HC and PwKOA during functional movement tasks according to the camera-based and the inertial sensor system. In addition, it was investigated whether there was a relationship between discriminating joint ROM and the individuals perceived degree of knee function (i.e. pain-related factors and disability). For the camera-based system, differences between HC and PwKOA were detected for all tasks, in all movement planes and for most joints (Table 3). However, it should be taken into account that for the present study the camera-based system was used as a reference, in order to determine for which angular waveforms the inertial sensor system had corresponding discriminating differences and to assess the waveform similarity (i.e. based on the coefficient of multiple correlation and root mean square error). For the corresponding differentiating parameters measured by the inertial sensor system, only those parameters were selected in which the difference in ROM was greater than the MDC and which had appropriate waveform similarity (more details in Additional files 1). As a result, discriminating differences between HC and PwKOA measured with inertial sensors were detected in all functional movements (except for the sit to stand task), but only for specific angular waveforms. Furthermore, significant correlations between discriminating joint motion of walking and the sideward lunge task and pain-related factors were identified, i.e. less ROM was related to increased perceived pain and fear of movement, respectively. No significant correlations were found between joint ROM and disability.

During walking, PwKOA had significantly less knee flexion ROM in both the stance and swing phase (0–33% and 49–92%) of the stride. Differences in the knee flexion ROM during the stance phase between HC and PwKOA were reported in previous studies, and were identified as a compensation strategy in order to reduce pain and dysfunction associated with the degenerative changes in the joint [34–36]. In addition, it was reported that PwKOA preserve a reduced knee flexion angle, i.e. stiffer knee, during push-off (around 60% of the stride) in order to decrease the loading on the knee joint, reduce pain and increase knee joint stability [34, 37]. In this view, the discriminating differences in the trunk and pelvic rotations might contribute as an adaptive movement strategy to reduce the step length [38–40], and subsequently reduce the cumulative loading on the knee joint [41, 42]. However, further research is required in order to establish this relationship.

The knee flexion ROM was not only reduced during walking but also for the other more physically demanding tasks. Where the HC had on average a similar knee flexion ROM during the forward lunge (54.5°), sideward lunge (59.1°) and walking (57.1°), PwKOA had a smaller and more variable knee flexion ROM in these tasks (44.1°, 41.9°, 54.8° respectively). Both groups increased their knee ROM in order to be able to ascent and descent the staircase (HC 63.1°, 78.6°; PwKOA 62.9°, 70.5°, respectively) and decreased their knee flexion ROM during the single leg squat (HC 44.1°; PwKOA 29.6). Although these tasks have a higher knee contact force compared to walking [43], the lower knee flexion ROMs were not necessarily associated with more pain, as only the knee flexion ROM during walking had a significant correlation with perceived pain (Table 4). Furthermore, no relation between KOOS function and knee flexion during gait, as already reported by Steultjens et al. (2000), or other tasks were found in this study [3]. In addition, within the present study only the reduced hip abduction ROM during sideward lunge was related to fear of movement. It is possible that persons with a higher level of fear of movement were adjusting the weight bearing over their painful knee by altering the hip abduction range of motion, in an attempt to unload the painful knee. A relation between joint kinematics in the adjacent joints of the painful joint and fear of movement is already earlier identified in persons with KOA. Hart et al. (2015) reported for example a correlation between higher fear of movement and higher trunk peak flexion during gait (r = 0.518, *p* = 0.02) [44].

Although it was hypothesised that (mal)adaptive movement strategies for PwKOA are more prominent during challenging and demanding tasks, the inertial sensor system was only able to measure differences between HC and PwKOA to a limited extend. Nevertheless, the results of this study suggest that during walking and more demanding tasks in which the knee contact force increases [43], PwKOA alter their movement patterns (i.e. especially knee flexion ROM), probably as an adaptive strategy to decrease the loading on the knee joint or to reduce pain [35]. Further research should investigate whether this strategy to reduce the loading on the knee joint is based on perceived pain and fear of movement during task execution itself, since there were no overall significant correlations found in this study between joint ROM, KOOS pain and TSK, which are measures assessing general pain and fear of movement.

While this study provides insights in the assessment of joint kinematics by means of an inertial sensor system during various functional tasks and the ability to discriminate HC from PwKOA, the results of this study should be interpreted with several limitations in mind. First of all, the sample size of the present study is relatively small in order to find a decent relationship between discriminating joint motion and the individuals perception on pain, disability and fear of movement. For the HC both legs were incorporated within the analysis, however no distinction was made in the analysis between the right of left leg. Furthermore, the significant difference in age between the KOA and control group (mean difference 5 years) might have influenced the results, and these limitations should be kept in mind when interpreting the results. Second, within the present study, persons with unilateral KOA were included but no distinction was made in the affected compartment (i.e. medial or lateral compartment) that was affected in the analysis, as there was not enough power to divide the patient sample in subgroups. As differences in alignment will alter the loading on the knee joint [45], this might induce different adaptive movement strategies related to pain. Moreover, as only participants with severe KOA (KL grade 3–4) were included, it might be expected that degenerative changes to the knee joint contribute to the reduction in knee flexion ROM. As the presence of osteophytes, bony enlargements and pain are associated with lower knee flexion ROM [46]. Since this study is part of a larger longitudinal study, in which persons with KOA were followed at multiple time points post-surgery, only participants on the waiting list for surgery (all KL grade IV, apart from one participant with KL grade III) were included. This limits the generalisation of the results to other populations or persons with KOA with different KL scores. Third, within the present study, only pain, disability and fear of movement were assessed. However, several other factors such as BMI, muscle strength, joint stability or self-efficacy, could have an effect on changes in joint kinematics and the development of (mal)adaptive movement strategies due to pain and disability [46–48]. When interpreting the results, it should furthermore be acknowledged that the number of practice trials were not registered but varied between participants and that the participants performed all tasks with barefoot. Finally, inertial sensors are easily disturbed by ferromagnetic materials, which subsequently negatively affect the estimation of the orientation and position of the sensor, and are required for the definition of the joint kinematics [49, 50]. Furthermore, the inertial system requires a static (N-pose) calibration, which assumes that the arms and legs are in full extension. As PwKOA experience difficulties to fully extend their legs and show malalignment of the knee joint, this will have an additional effect on the accuracy of the measures by the inertial sensor system [51]. New methods that overcome these constraints by performing a dynamic calibration and track motion without using the magnetometer have been developed [52, 53], but these methods need further development before they can be implemented in clinical research. Nevertheless, the strength of this study is that, based on inertial sensor measurements, several clinically relevant tasks were reported that were reliable and valid, that discriminated between HC and PwKOA, and can be measured outside the laboratory setting. This enables the implementation of the joint motion assessment in clinical practice. Moreover, in a longitudinal fashion, it provides the opportunity to assess the progression of KOA, the development of (mal)adaptive movement strategies and the effect of therapy.

## Conclusion

The camera-based system discriminated between HC and PwKOA in all tasks and in all movement planes. While the inertial sensor technology was able to evaluate that PwKOA walk with less trunk and pelvic rotation and less knee flexion. Reduced knee flexion ROM was related to perceived pain as measured with PROMs. Limited knee flexion ROM in comparison with HC was also observed during the execution of more challenging and demanding tasks such as the forward lunge, sideward lunge, single leg squat and ascending and descending stairs. Additionally, during the sideward lunge task, PwKOA have less ankle plantar flexion and less hip abduction. Reduced hip abduction ROM was associated with a higher level of fear of movement. Further research should focus on including larger groups of PwKOA, and to differentiate between medial and lateral KOA during the execution of physically demanding tasks and evaluate the associations with pain and disability related to movement. Although, the inertial sensor system found discriminating differences in joint motion between PwKOA and HC, the technology could be improved in such way that small movement deviations in clinically relevant tasks.

## Supplementary information

**Additional file 1.**

## Data Availability

The datasets used and/or analysed during the current study are available from the corresponding author on reasonable request.

## Abbreviations

PwKOA: Persons with knee osteoarthritis
HC: Healthy controls
SPM1D: One-dimensional statistical parametric mapping
ROM: Range of motion
PROMs: Patient reported outcome measures
KOA: Knee osteoarthritis
DOF: Degrees of freedom
KOOS: Knee injury and Osteoarthritis Outcome Score
ADL: Function in daily living
Sports/Rec: Function in sports and daily living
QOL: Knee related quality of life
TSK: TAMPA scale for Kinesiophobia
