# Supplementary material for: Functional movement assessment by means of inertial sensor technology to discriminate between movement behaviour of healthy controls and persons with knee osteoarthritis
Source: J Neuroeng Rehabil. 2020 May 19;17:65. doi: 10.1186/s12984-020-00694-2 (PMC7236325; doi:10.1186/s12984-020-00694-2)
Supplement: Supplementary file 1 — Additional file 1. [file 12984_2020_694_MOESM1_ESM.docx]

**Additional files 1:**

**Reliability, Agreement and Construct validity of discriminating parameters.**

**Background**

Assessment of the psychometric properties of the joint kinematics assessed by means of an inertial sensor system is important, as they are task specific. Previously, we have investigated the reliability and agreement of functional movement tasks, included within the present study, from distinct points within the waveform (i.e. min, max, rom) and from distinct movement phases such as the swing and stance phase [1, 2]. However, these were not sufficient, as for the present study the entire waveform was evaluated, in order to determine which joint kinematics differentiate between HC and PwKOA. Additionally, to determine if differences between HC and PwKOA were greater than the measurement error of the inertial sensor technology, the minimum detectable change (MDC) was determined, based on the ROM of the entire waveform. Finally, differences can only be interpreted when they were reliable and valid.

**Objective**

To investigate the within-session reliability, agreement and construct validity to determine the reproducibility and accuracy of the discriminating parameters.

**Methods**

To evaluate the reliability and agreement of the joint kinematics measured by means of inertial sensors, 20 HC were included. This was a conscious decision, as day-to-day movement execution of patients might be affected through pain or stiffness. More details about the procedures are described elsewhere [1, 2]. For evaluation of the construct validity, only twelve HC participated (the same as in the present study), as both legs were included for analysis, and these data were pooled with the affected leg of 19 PwKOA in order to evaluate the waveform similarity between the waveforms from the camera-based system and the inertial sensor system.

Reliability and agreement were determined based on joint ROM, which was calculated as the absolute difference between the minimum and maximum angle of the normalized waveforms. Intraclass correlation coefficients (ICC), including the 95% confidence interval were determined using SPSS (version 25, IBM Corporation, Amonk, NY). Single data was used to calculate the within-session reliability (ICC2,1) and agreement. Average data of four repetitions was used to calculate the between-session and between-operator reliability (ICC2,k) and agreement. ICCs ≥ 0.90 were considered as excellent, 0.70–0.89 good, 0.69–0.40 acceptable, and <0.40 as low. Agreement was determined based on the standard error of the measurement (SEM), based on the square root of the mean square error term of the analysis of variance (ANOVA) and the minimum detectable change (MDC) between two sessions, using the SEM (MDC = SEM × 1.96 × √2). To provide information on the magnitude of the SEM with respect to the ROM, a proportional SEM (%SEM) was calculated (%SEM = (SEM/mean)*100%).

Construct validity was determined by comparing the similarity between angular waveforms from the camera-based system and inertial sensor system. Waveform similarity was obtained using the root mean squared error (RMSE) and the coefficient of multiple correlation (CMC) [3]. With respect to the CMC, in case the offset between two waveforms is comparable, the CMC returns “not a real number” (NAN). Therefore, the CMC was calculated after offset removal [3]. For further interpretation, the amount of NaNs and the mean and standard deviation (SD) are presented. The CMCs were interpreted as follows: CMC >0.95 excellent, 0.85-0.94 very good, 0.75-0.84 good, 0.65-0.74 moderate and CMCs <0.64 low.

**Results**

Good to excellent within-session reliability was observed for the discriminating joint kinematics during walking, FL, SL, SLS and ascending stairs (ICC 0.73-0.95). Acceptable reliability was found for descending stairs (ICC 0.59). The agreement was generally good, with SEMs ranging between 1.06 and 4.44 degrees for all tasks (Table A1). The proportional SEM (%SEM) was generally small as well, except for the pelvis in/external rotation ROM during walking (%SEM 15.9%) and the knee flexion/extension ROM during the SLS (%SEM 11.3) which had a larger %SEMs.

The CMCs (i.e. waveform similarity) of the discriminating parameters ranged from 0.86-1.00 and the RMSE from 1.56-4.94° (Table A1). Despite the fact that CMCs were calculated based on the corrected data, it was impossible to include all the recorded trials [3]. For walking, it was not possible to calculate the CMC for the trunk and pelvic in/external rotation angles of 9 and 10% of the data, respectively.

**Conclusion**

For all discriminating joint angles in all tasks, the within-session reliability was good to excellent, except for the knee flexion ROM during descending stairs which had moderate reliability. The construct validity of discriminating joint motion was very good to excellent for all tasks, with RMSE below five degrees.

**References**

1. R. van der Straaten, A.K.B.D. Bruijnes, B. Vanwanseele, I. Jonkers, L. De Baets, A. Timmermans, Reliability and Agreement of 3D Trunk and Lower Extremity Movement Analysis by Means of Inertial Sensor Technology for Unipodal and Bipodal Tasks, Sensors (Basel, Switzerland). 19 (1) (2019) 141.

2. R. van der Straaten, A. Timmermans, A. Bruijnes, B. Vanwanseele, I. Jonkers, L. De Baets, Reliability of 3D Lower Extremity Movement Analysis by Means of Inertial Sensor Technology during Transitional Tasks, Sensors. 18 (8) (2018) 2638.

3. A. Ferrari, A.G. Cutti, A. Cappello, A new formulation of the coefficient of multiple correlation to assess the similarity of waveforms measured synchronously by different motion analysis protocols, Gait & posture. 31 (4) (2010) 540-2.

Table A1: Construct validity and within-session Reliability and Agreement from discriminating joint kinematics

| **Task** | **joint** | **%NaN** | **CMC** | **RMSE** | **ICC** | **CI** | **mean (± SD)** | **SEM** | **MDC** | **%SEM** | **%MDC** |
| --- | --- | --- | --- | --- | --- | --- | --- | --- | --- | --- | --- |
| walk | Trunk  in/ext. rotation | 9 | 0,86 (± 0,19) | 4,63 (± 4,09) | 0,95 | 0,90 - 0,98 | 14,6 (± 4,6) | 1,06 | 2,94 | 7,3 | 20,2 |
|  | Pelvis  in/ext. rotation | 10 | 0,91 (± 0,12) | 2,46 (± 2,24) | 0,81 | 0,65 - 0,91 | 10,7 (± 3,8) | 1,70 | 4,72 | 15,9 | 44,1 |
|  | Knee  flex/extension | 0 | 0,99 (± 0,01) | 3,37 (± 1,32) | 0,85 | 0,73 - 0,93 | 64,9 (± 3,2) | 1,28 | 3,56 | 2,0 | 5,5 |
| FL | Knee  flex/extension | 0 | 0,99 (± 0,01) | 2,92 (± 1,76) | 0,92 | 0,83 - 0,97 | 69,9 (± 11,3) | 3,28 | 9,09 | 4,7 | 13,0 |
| SL | Hip  ab/adduction | 0 | 0,95 (± 0,06) | 2,75 (± 1,25) | 0,87 | 0,76 - 0,94 | 37,3 (± 5,0) | 1,74 | 4,83 | 4,7 | 12,9 |
|  | Knee  flex/extension | 0 | 0,99 (± 0,01) | 2,69 (± 1,05) | 0,91 | 0,83 - 0,96 | 67,1 (± 10,8) | 3,35 | 9,28 | 5,0 | 13,8 |
|  | Ankle  flex/extension | 0 | 0,96 (± 0,14) | 4,94 (± 2,88) | 0,92 | 0,85 - 0,96 | 66,6 (± 10,1) | 2,81 | 7,79 | 4,2 | 11,7 |
| SLS | Knee  flex/extension | 0 | 1,00 (± 0,01) | 1,56 (± 1,20) | 0,73 | 0,56 - 0,87 | 39,2 (± 8,7) | 4,44 | 12,31 | 11,3 | 31,4 |
| Ascending stairs | Knee  flex/extension | 0 | 0,99 (± 0,01) | 2,89 (± 1,82) | 0,90 | 0,79 - 0,96 | 90,8 (± 6,2) | 2,18 | 6,04 | 2,4 | 6,7 |
| Descending stairs | Knee  flex/extension | 0 | 1,00 (± 0,01) | 3,05 (± 1,74) | 0,59 | 0,36 - 0,80 | 82,2 (± 5,3) | 3,20 | 8,86 | 3,9 | 10,7 |
